# A Comparative Study of Dietary Intake, Nutritional Status, and Frailty in Outpatients and Inpatients with Liver Cirrhosis

**DOI:** 10.3390/nu17030580

**Published:** 2025-02-05

**Authors:** Saniya Khan, Sara Sansoni, Simone Di Cola, Lucia Lapenna, Manuela Merli

**Affiliations:** Department of Translational and Precision Medicine, Sapienza University of Rome, 00185 Rome, Italy; saniya.khan@uniroma1.it (S.K.); sansoni.1855402@studenti.uniroma1.it (S.S.); simone.dicola@uniroma1.it (S.D.C.); lucia.lapenna@uniroma1.it (L.L.)

**Keywords:** liver cirrhosis, frailty, dietary intake, nutrition

## Abstract

**Background:** Liver cirrhosis is associated with significant nutritional challenges, including malnutrition, sarcopenia, and frailty, which impact clinical outcomes. The severity of these issues may vary between inpatient and outpatient settings, but there is a limited understanding of how these conditions manifest in these populations. This study aims to compare the nutritional status, dietary intake, and frailty in outpatients and inpatients with liver cirrhosis and to explore potential sex-specific differences. **Methods:** This prospective observational study enrolled 195 patients with liver cirrhosis from the Gastroenterology ward and Outpatient Clinic of Policlinico Umberto I, Sapienza University of Rome, between May 2023 and July 2024. Nutritional status was assessed using anthropometric measurements, dietary recall, and food frequency questionnaires. Sarcopenia was evaluated using the SARC-F questionnaire and handgrip strength. Frailty was assessed using the Liver Frailty Index (LFI). Data on clinical characteristics, comorbidities, and disease severity were also recorded. **Results:** The inpatient group (*n* = 69) had significantly lower BMI, mid-upper arm circumference, and triceps skinfold compared to outpatients (*n* = 126). Inpatients exhibited higher frailty, with 73.9% classified as frail according to the LFI, compared to 39.6% in outpatients (*p* < 0.001). Dietary intake revealed that 91% of inpatients had an energy intake deficit compared to 76% of outpatients (*p* = 0.009). Protein intake was inadequate in 84% of inpatients versus 61% of outpatients (*p* < 0.001). Sex-specific analysis showed that females had a higher prevalence of sarcopenia than males (64.4% vs. 38.2%, *p* < 0.001) and experienced more significant protein deficits (74.3% vs. 57.6%, *p* = 0.021). Females also had higher LFI score (4.77 ± 0.88 vs. 4.45 ± 0.91, *p* = 0.034). Multivariate analysis showed that CTP, LFI, and protein deficit are independently associated with hospitalization. **Conclusions:** Inpatients with liver cirrhosis are at higher risk for malnutrition, frailty, and inadequate nutrient intake compared to outpatients, emphasizing the need for targeted nutritional interventions in hospital settings. Additionally, females with cirrhosis are more prone to sarcopenia and frailty, requiring gender-specific approaches to nutrition.

## 1. Introduction

Liver cirrhosis is a chronic and progressive disease that significantly impairs liver function, often leading to complications such as malnutrition, sarcopenia, and frailty, with these issues becoming more pronounced as the disease progresses [1,2]. These nutritional challenges are critical factors in the clinical management of cirrhotic patients, as they can exacerbate disease progression, increase susceptibility to infections, and decrease quality of life [3,4].

Malnutrition in liver cirrhosis is multifactorial, stemming from altered nutrient metabolism, poor dietary intake, increased energy expenditure, and malabsorption of nutrients [5,6]. Furthermore, systemic inflammation and hypermetabolism associated with liver disease contribute to muscle protein breakdown, leading to sarcopenia—a key component of malnutrition in these patients. Both inpatient and outpatient populations with cirrhosis face these challenges, but the degree to which nutritional status and dietary intake are affected may vary significantly depending on the care setting and severity of the disease [7]. Additionally, cirrhotic patients commonly experience micronutrient alterations, including deficiencies in vitamins and minerals, and this may further contribute to patients’ impairment [8,9].

Inpatients with liver cirrhosis, who often present with decompensated disease, are at heightened risk for rapid nutritional decline due to prolonged hospitalization, restricted dietary intake, and acute complications such as gastrointestinal bleeding, ascites, and hepatic encephalopathy (HE) [10,11]. In contrast, outpatients, although generally more stable, continue to experience dietary deficiencies that can impact their long-term prognosis. Frailty, a condition of reduced physiological reserve and increased vulnerability to adverse outcomes, further complicates this landscape [10,12]. The Liver Frailty Index (LFI) offers an objective measure of frailty and is increasingly recognized as a key determinant of prognosis in cirrhosis [13,14]. Frailty, in conjunction with malnutrition and sarcopenia, has been linked to worse clinical outcomes, increased risk of complications, and reduced transplant eligibility in this population [1].

Despite the known risks of malnutrition and frailty, there remains a limited understanding of how these issues manifest differently in inpatient versus outpatient populations. At the same time, several studies have evaluated the nutritional status of cirrhotic patients, whether as outpatients [15,16,17,18] or inpatients [19,20,21,22]; there is a notable gap in data comparing these two groups. In addition, very few studies have investigated dietary habits and dietary intake levels in cirrhotic patients [23,24,25,26,27], and no study, to our knowledge, has evaluated dietary intake according to the severity of liver disease and the patient setting. This knowledge is relevant to addressing the appropriate dietary counseling and providing sufficient calories and adequate high-quality proteins based on their disease severity, care setting, and individual characteristics. This study aims to provide a comprehensive overview of the nutritional status of cirrhotic patients, their dietary consumption of macronutrients, and frailty in two different clinical settings and highlight any disparities between inpatients and outpatients. We also explored possible sex-related differences in nutritional status.

## 2. Materials and Methods

### 2.1. Study Population

In this prospective observational study, all adult patients with liver cirrhosis attending the Outpatient Clinic or admitted to the Gastroenterology ward of Policlinico Umberto I, Sapienza University of Rome, between May 2023 and July 2024 underwent a nutritional screening and were enrolled in the study. The exclusion criteria were: (1) the patient’s refusal to undergo the evaluation; (2) the inability of the patient to undergo a complete evaluation due to severe clinical conditions during hospitalization (like chronic renal failure requiring hemodialysis, severe heart disease, a severe chronic pulmonary disease requiring continuous oxygen therapy, etc.); (3) patients whose cirrhosis status was unclear; (4) patients with a prior liver transplant; (5) elective admission for only day procedures (paracentesis or endoscopy), and (6) hepatocellular carcinoma (HCC) out of Milan criteria.

### 2.2. Ethical Approval

The study was approved by the Ethical Committee of the Sapienza University of Rome (Prot. 0057/2023 approved on 26 January 2023). Informed consent was obtained from all participants after an extensive explanation of the relevance of nutrition and lifestyle in chronic liver diseases.

### 2.3. Data Collection

Demographic and clinical information, including sex, age, etiology of cirrhosis, previous clinical history, relevant comorbidities, MELD (model for end-stage liver disease) score, MELD-Na (MELD with sodium) score, and Child–Turcotte–Pugh (CTP) score, etc., were recorded from hospital records at patient’s enrolment.

### 2.4. Nutritional Assessment

For inpatients, body weight was noted on the first and last day of hospitalization to evaluate weight changes.

Body mass index (BMI) was calculated by using a standard formula in which the estimated dry weight was utilized when needed. Dry weight was estimated in the case of fluid retention as follows: a reduction of 5% for mild ascites, 10% for moderate ascites, and 15% for tense ascites. Additionally, a further 5% was subtracted for the presence of pedal edema [28].

Dietary habits were analyzed through 24-h dietary recall (24 HR) and food frequency questionnaire (FFQ). These methods were used to evaluate the frequency of consumption of food categories and to calculate the daily energy, protein, carbohydrate, and fat consumption. Energy and protein deficits were calculated based on the daily energy (35 kcal/kg body weight/day) and protein (1.2 g/kg body weight/day) requirements, as recommended by the EASL Clinical Practice Guidelines on Nutrition in Chronic Liver Disease [8]. The energy and protein deficits were determined by calculating the difference between each patient’s actual daily intake and the recommended intake for both nutrients.

### 2.5. Physical and Muscle Mass Assessment

Mid-upper arm circumference (MUAC) was measured with a non-stretchable tape with an accuracy of 0.1 cm. Triceps skinfold (TSF) thickness was measured using Harpenden calipers with an accuracy of 1 mm at the mid-point between the acromion and the olecranon processes [29]. All the measurements were taken on the non-dominant arm. The mid-arm muscle circumference (MAMC) was calculated using a standard formula based on MUAC and TSF [30].

Handgrip strength (HGS) was measured in kilograms using a handheld dynamometer in the dominant hand, with an average of three trials used for analysis [31]. Low muscle function was defined as HGS < 27 kg in males and <16 kg in females [32]. Physical performance was further assessed using a balance test, where participants balanced for up to 10 s in three positions (side-by-side, semi-tandem, and tandem), and the chair stand test (CST), which timed the completion of five chair stands with arms folded across the chest [33]. Using HGS, the balance test, and CST results, the LFI was calculated. Frailty was classified as robust (LFI < 3.2), prefrail (LFI 3.2–4.4), or frail (LFI ≥ 4.5) [34].

The SARC-F is a bedside tool consisting of five items: (1) strength, (2) walking, (3) rising from a chair, (4) climbing stairs, and (5) falls. Each item is scored from 0 (best) to 2 (worst), with a total score ranging from 0 to 10 points. A score of 4 or higher (SARC-F ≥ 4) indicates an increased risk of sarcopenia [35].

### 2.6. Statistical Analysis

Statistical analysis was performed using SPSS software (version 28.0.1.1). Data were expressed as mean ± standard deviation (SD) or median (range) in the case of continuous variables and as number (percentages) for categorical variables. Independent sample *t*-test or Mann–Whitney and Chi-Square test were used for group comparisons. A *p*-value < 0.05 was considered statistically significant.

## 3. Results

### 3.1. Demographic and Clinical Characteristics of Patients

One hundred and ninety-five patients with liver cirrhosis were enrolled in the study. Males were prevalent (69.7%), and alcohol was the most common etiology (43.6%). A concomitant HCC within Milan criteria was present in 12% of patients. The child score showed that half of the patients belonged to the Child A class. The median MELD score was 12, ranging from 6 to 27 (Table 1). BMI ranged from 14.8 to 47.6 kg/m^2^. Frailty was reported in 51.8% of patients, while 45.6% were categorized as pre-frail. According to SARC-F (≥4), 46.2% of the patients showed signs of sarcopenia (Table 2).

**Figure 1:**
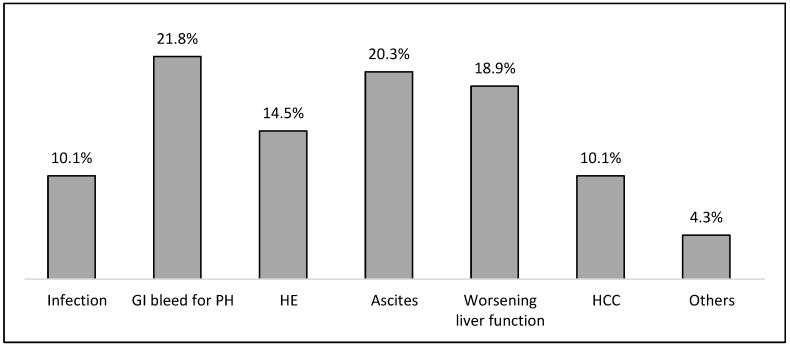
Reasons for hospitalization in 69 patients with liver cirrhosis.

Of the 195 patients included in the study, 126 (64.6%) were outpatients, while 69 (35.4%) were inpatients. Age and sex were comparable between the two groups. However, disease severity scores- such as MELD, MELD-Na, and CTP were significantly higher in inpatients compared to outpatients. The most common cause of hospitalization among inpatients was GI bleeding for portal hypertension (PH), followed by ascites and worsening liver functions, as shown in Figure 1. Approximately half of the patients with GI bleeding for PH were hospitalized with active bleeding, and half for treating a recent bleeding episode by endoscopic banding. The mean length of hospitalization was 10.97 ± 12.88 days, and the mean dry weight loss during the hospital stay was 3.96 ± 4.78 kg.

Regarding the evaluation of dietary habits, FFQ highlighted that 92% of the subjects took at least one protein source per day, 74% consumed a portion of milk and milk products per day. Although most patients consumed at least one protein source per day, only 32% achieved the recommended daily protein intake. Furthermore, 81% of the patients had an energy intake lower than recommended. When investigated, 90.8% of the patients reported having a good appetite, highlighting the lack of knowledge among cirrhotic patients about the dietary recommendations specific to their disease condition.

### 3.2. Comparison of Nutritional Status and Frailty Between Inpatients and Outpatients

Overall, the in-hospital population, being more severely affected due to decompensated cirrhosis, exhibited a poorer nutritional status. BMI was significantly lower in inpatients as compared to outpatients (24.83 ± 5.01 vs. 27.45 ± 6.44; *p* = 0.002). Additionally, other anthropometric measurements, such as MUAC and TSF, were also significantly lower in inpatients compared to outpatients, respectively 29.39 cm vs. 32.01 cm (*p* = 0.014), and 13.10 mm vs. 16.56 mm (*p* = 0.014).

Regarding sarcopenia, as assessed by the SARC-F questionnaire, 42% of outpatients and 47% of inpatients were identified as being at an increased risk of sarcopenic. However, the difference between these groups was not statistically significant.

LFI score revealed a significantly higher prevalence of frailty in inpatients (73.9% vs. 39.6%; *p* < 0.001). Furthermore, inpatients had a higher mean LFI score as compared to outpatients (*p* < 0.001). HGS, an important indicator of muscle function, was also significantly lower in inpatients (22.00 ± 13.44 kg vs. 25.13 ± 10.72 kg, *p* = 0.04).

### 3.3. Comparison of Dietary Intake Between Inpatients and Outpatients

**Figure 2:**
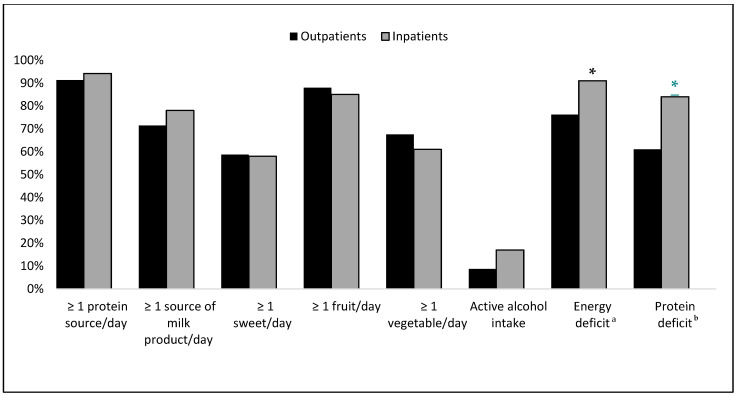
Dietary habits of 195 patients with liver cirrhosis, classified according to patient setting and consumption of nutrients ^a–b^, * *p* < 0.05. The frequency of consumption of a particular food group or nutrient by patients was estimated using food frequency questionnaire data and expressed as percentages. ^a^ The presence of an energy deficit was calculated based on the number of patients whose actual daily energy intake was lower than the recommended energy intake. ^b^ The presence of a protein deficit was calculated based on the number of patients whose actual daily protein intake was lower than the recommended protein intake.

Figure 2 shows the results from the evaluation of the patient’s eating habits. FFQ revealed that a majority of patients, both outpatients and inpatients, consumed at least one protein source daily, including meat, fish, eggs, or legumes. Specifically, 91% of outpatients and 94% of inpatients met this criterion. Additionally, 71% of outpatients and 78% of inpatients consumed at least one serving of milk or milk products per day. Regarding fruit consumption, 85% of inpatients and 88% of outpatients reported eating at least one serving of fruit daily. For vegetable intake, 60% of inpatients and 67% of outpatients consumed at least one serving per day. Notably, none of these differences in dietary habits between outpatients and inpatients were statistically significant.

Table 3 compares the energy and protein intake against the recommended guidelines, significant findings emerged. Among outpatients, 76% had an energy intake deficit, while this figure was significantly higher at 91% among inpatients (*p* = 0.009). The remaining 24% of outpatients and 9% of inpatients either met or exceeded the recommended energy intake. 

Although the average daily energy intake (kcal/kg/day) was slightly higher in outpatients compared to inpatients (23.2 kcal vs. 21.4 kcal), this difference was not statistically significant (*p* = 0.089). However, a significantly higher proportion of inpatients (91%) had an energy intake deficit compared to outpatients (76%), with this difference being statistically significant (*p* = 0.009). 

Similarly, the daily protein intake was comparable between the two groups, with outpatients consuming an average of 1.0 ± 0.36 g/kg/day and inpatients consuming 0.93 ± 0.31 g/kg/day (*p* = 0.183). However, the proportion of patients with a protein intake deficit was significantly different, with 84% of inpatients falling below the recommended intake, compared to 61% of outpatients (*p* < 0.001). 

### 3.4. Sex-Specific Clinical and Demographic Characteristics

There was no difference in mean age between males and females (63.17 ± 10.17 years vs. 64.36 ± 10.38 years, *p* = 0.458). A significantly higher proportion of males had alcohol-related cirrhosis (48.5% vs. 32.2%, *p* = 0.001). The presence of ascites, varices, HE, HCC, and co-morbidities showed no significant differences between males and females. The MELD and CTP scores classifications were also comparable between the two groups, with no significant differences across these variables (Table 4).

### 3.5. Comparison of Nutritional Status, Frailty, and Dietary Intake Between Male and Female Patients

As shown in Table 5, BMI was similar between the two groups, with males averaging 26.27 ± 5.43 kg/m² and females 26.91 ± 7.58 kg/m² (*p* = 0.559). While the distribution of patients across BMI categories differed slightly, with females having a higher percentage of underweight and obese individuals, this difference was not statistically significant (*p* = 0.083). However, females had significantly higher TSF than males (19.81 mm vs. 14 mm, *p* = 0.002). The SARC-F questionnaire identified a higher proportion of females at increased risk of sarcopenia compared to males (64.4% vs. 38.2%; *p* < 0.001). HGS was also significantly lower in females (15.4 ± 7.63 kg vs. 27.82 ± 11.3 kg; *p* < 0.001). Low muscle function was more common in females (61% vs. 43.4%; *p* = 0.024). Moreover, females had a significantly higher LFI as compared to males (4.77 ± 0.88 vs. 4.45 ± 0.9; *p* = 0.034).

Table 6 reports dietary intake according to the sex. Mean energy intake did not differ between males and females (*p* = 0.91). Besides, males experienced a higher energy deficit than females (86.8% vs. 69.5%; *p* = 0.004). Mean protein intake was also comparably lower than the recommended daily protein intake in males and females. The protein deficit was more common among males than in females (74.3% vs. 57.6%; *p* = 0.021), indicating significant sex-specific nutritional differences.

### 3.6. Factors Associated with Hospitalization in Liver Cirrhosis Patients

In univariate analysis, the presence of HCC (*p* < 0.001), high MELD score (*p* = 0.001), MELD-Na score (*p* < 0.001) and CTP score (*p* < 0.001), low BMI (*p* = 0.003), MUAC (*p* = 0.017), TSF (*p* = 0.038), high LFI (*p* < 0.001), protein deficit (*p* = 0.01), and energy deficit (*p* = 0.012) were associated with hospitalization, as shown in Table 7. However, on multivariate analysis, only CTP (*p* = 0.01), LFI (*p* = 0.011), and protein deficit (*p* = 0.031) scores were found to be independently associated with hospitalization.

## 4. Discussion

This study presents an in-depth comparison of the nutritional status, frailty, and dietary habits of inpatients versus outpatients with liver cirrhosis, revealing nutritional vulnerabilities across both settings. Inpatients, in particular, exhibited compromised nutritional status, greater frailty, and more significant dietary intake deficits, highlighting the importance of tailored nutritional support strategies for each group. To our knowledge, this is the first study comparing the disparities between these two patient settings.

As anticipated, disease severity scores- such as MELD, MELD-Na, and CTP were significantly higher in inpatients, reflecting more advanced liver disease and poorer prognosis. A higher prevalence of CTP C patients, associated with the highest risk of mortality, was observed among inpatients. These findings emphasize the expected, more severe clinical condition of hospitalized patients. Moreover, multivariate binary logistic regression analysis showed that CTP score, LFI, and presence of protein deficit were independently associated with hospitalization.

Frailty and reduced muscle function were markedly more prevalent among inpatients, correlating with advanced disease and lower functional status, indicating vulnerability to adverse health outcomes. Although the rates of increased risk of sarcopenia are comparable across both groups, the consistently high levels emphasize the need for sarcopenia screening and intervention in all patients, as muscle deterioration directly impacts quality of life and outcomes.

While average daily energy and protein intakes were comparable between groups, inpatients had significantly higher percentages of energy and protein deficits. Most inpatients were unable to meet recommended daily energy requirements, indicating an urgent need for enhanced nutritional support during hospitalization, including prolonged fasting for medical investigations. Protein intake deficiencies were widespread in inpatients, with only a fraction meeting the recommended intake. This shortfall in protein intake is concerning, given its critical role in preserving muscle mass and preventing further deterioration in patients already prone to muscle wasting [36,37]. Addressing protein intake is essential to prevent accelerated nutritional decline and manage sarcopenia in patients with advanced liver disease [8,38].

Inpatients had significantly lower anthropometric measurements compared to outpatients. These findings reinforce that inpatients are at much greater risk for nutritional deficiencies. Altered body composition measures, indicative of muscle and fat loss, are attributable to increased metabolic demands, inflammation, and altered nutrient metabolism, all compounding the risk of malnutrition [1,5].

The study’s findings emphasize the need for tailored nutritional interventions across different care settings for liver cirrhosis patients. Outpatients, despite being in a more stable phase of their condition, still exhibit significant dietary deficiencies, pointing out that nutritional impairments in liver cirrhosis can occur even in the absence of severe disease complications. Inpatients, on the other hand, experience rapid nutritional decline and prolonged periods of inadequate intake, requiring intensified support strategies, such as enteral or parenteral nutrition, to meet their elevated metabolic needs and reduce the adverse effects of malnutrition. Nutritional strategies should include frequent assessments and adjustments to align with each patient’s fluctuating clinical status using a multidisciplinary team to ensure better outcomes [39], and it might improve patient adherence.

Furthermore, this study highlights significant sex-specific disparities in nutritional vulnerabilities, with female patients showing a higher prevalence of sarcopenia and lower muscle function compared to males. This suggests that females with liver cirrhosis may require sex-specific nutritional approaches, as they are at greater risk for muscle deterioration and frailty.

While this study provides valuable insights, it is limited by its single-center design, which may affect the generalizability of the findings. The tool used to assess dietary intake relies on self-reported data, which is subjective and susceptible to recall bias [40,41]. Factors such as patients’ memory, comprehension, and willingness to report accurately may affect the reliability and validity of the data. Furthermore, information on micronutrients, including vitamins and minerals, is not available in this study cohort. The 24-h dietary recall and food frequency questionnaires are not precise methods for assessing micronutrient intake, as they do not account for potential ongoing supplementation. Due to these limitations, the evaluation of micronutrient intake was excluded during the protocol design. Moreover, while objective measures like frailty scores and muscle strength assessments provided reliable indicators of patient status, incorporating additional assessments of quality of life and functional ability would offer a more comprehensive understanding of the patient experience.

Future studies should explore strategies to improve dietary intake in patients with liver cirrhosis, specifically addressing energy and protein deficits. The involvement of a multidisciplinary team, including dietitians, physicians, and physical therapists, could be a more effective approach to providing tailored counseling and interventions. This collaboration can help patients understand the importance of nutrient intake, improve adherence to dietary recommendations, and better manage their nutritional status for improved outcomes. Additionally, longitudinal studies assessing the progression of nutritional status, muscle function, and frailty over time could offer deeper insights. Integrating quality-of-life measures and patient-reported outcomes could further enhance our understanding of the broader implications of nutritional interventions.

## 5. Conclusions

In conclusion, this study identifies substantial nutritional vulnerabilities in liver cirrhosis patients, with significant differences between inpatients and outpatients. Inpatients, who often have more advanced disease, had a pooper nutritional status, higher frailty, and more severe energy and protein deficits as compared to the outpatient population. Additionally, sex-specific differences were observed, with female patients exhibiting a higher prevalence of sarcopenia and frailty and also higher protein and energy deficits as compared to male patients with liver cirrhosis. There is a need for targeted nutritional strategies tailored to clinical settings and patient demographics, particularly focusing on adequate dietary intake and strategies to manage frailty. By implementing individualized nutritional assessments and targeted interventions, healthcare providers can address the complex and challenging nutritional needs of liver cirrhosis patients, ultimately improving prognosis and alleviating the burden of malnutrition in this vulnerable population.

## Figures and Tables

**Table 1:** Demographics and clinical characteristics of all patients with liver cirrhosis.

Variable	All (*n* = 195)	Outpatients (*n* = 126)	Inpatients (*n* = 69)	*p* Value
Age (years)	63.53 ± 10.23	64.47 ± 9.54	61.81 ± 11.21	0.083
Sex				0.348
Male	136 (69.7)	85 (67.5)	51 (73.9)	
Female	59 (30.3)	41 (32.5)	18 (26.1)	
Etiology				0.767
Alcohol	85 (43.6)	58 (46.1)	27 (39.1)	
Metabolic	40 (20.5)	26 (20.6)	14 (20.3)	
Viral	55 (28.2)	33 (26.2)	22 (31.9)	
Others	15 (7.7)	9 (7.1)	6 (8.7)	
Ascites	66 (33.8)	32 (25.4)	34 (49.3)	<0.001
Varices	77 (39.5)	49 (38.8)	28 (40.6)	0.817
HE	8 (4.1)	2 (1.6)	6 (8.7)	0.017
HCC	24 (12.3)	3 (2.4)	21 (30.4)	<0.001
Presence of co-morbidities	127 (65.1)	83 (65.9)	44 (63.8)	0.768
INR	1.27 ± 0.27	1.19 ± 0.13	1.76 ± 0.39	0.008
Total protein (g/dL)	7.28 ± 0.76	7.42 ± 0.69	6.2 ± 0.29	<0.001
Serum albumin (g/dL)	3.78 ± 0.59	3.93 ± 0.49	2.95 ± 0.4	<0.001
MELD score	12.49 ± 4.59	10.96 ± 3.55	15.12 ± 5	<0.001
MELD > 15	57(29.2)	23 (18.3)	34 (49.3)	<0.001
MELD-Na score	14.18 ± 5.46	12.17 ± 4.28	17.25 ± 5.68	<0.001
CTP score	7.01 ± 1.8	6.28 ± 1.33	8.26 ± 1.83	<0.001
Child–Pugh class	A: 97 (49.8)	A: 81 (64.3)	A: 16 (23.2)	<0.001
	B: 80 (41.0)	B: 43 (34.1)	B: 37 (53.6)	
	C: 18 (9.2)	C: 2 (1.6)	C: 16 (23.2)	

Data presented as mean ± SD and median (range) or as n of patients and percentages (%). Abbreviations: HE—hepatic encephalopathy; HCC—hepato-cellular carcinoma; MELD—model for end-stage liver disease; INR—international normalized ratio; MELD-Na—model for end-stage liver disease-sodium; CTP—Child–Turcotte–Pugh.

**Table 2:** Anthropometry measurements and frailty in patients with liver cirrhosis.

Variable	All (*n* = 195)	Outpatients (*n* = 126)	Inpatients (*n* = 69)	*p* Value
BMI (kg/m^2^)	26.48 ± 6.07	27.45 ± 6.44	24.83 ± 5.01	0.002
<18.9 kg/m^2^	12 (6.2)	4 (3.2)	8 (11.6)	
18.9–24.9 kg/m^2^	77 (39.5)	46 (36.5)	31 (44.9)	
>24.9–29.9 kg/m^2^	56 (28.7)	34 (27.0)	22 (31.9)	
>29.9 kg/m^2^	50 (25.6)	42 (33.3)	8 (11.6)	
MUAC (cm)	31.24 ± 5.49	32.01 ± 5.44	29.39 ± 5.21	0.014
TSF (mm)	15.61 ± 8.07	14.8 (4–39.9)	12.2 (4.2–36.2)	0.014
MAMC (mm)	245.97 (175.8–370.68)	203.99 (175.8–370.68)	164.47 (188.25–322.17)	0.056
Sarcopenia (SARC-F score ≥ 4)	90 (46.2)	53 (42.1)	33 (47.8)	0.729
HGS (kg)	24.05 ± 11.79	25.13 ± 10.72	22 ± 13.44	0.04
Low muscle function	95 (48.7)	57 (45.2)	38 (55.1)	0.189
CST (s)	12.26 (4.18–40)	12.3 (4.18–40)	12.95 (7–32.8)	0.208
LFI score	5.44 ± 0.91	4.33 ± 0.69	5.01 ± 1.15	<0.001
Frail patients (LFI ≥ 4.5)	101 (51.8)	50 (39.6)	51 (73.9)	<0.001

Data presented as mean ± SD and median (range) or as n of patients and percentages (%). Abbreviations: BMI—body mass index; MUAC—mid-upper arm circumference; TSF—triceps skinfold; MAMC—mid-arm muscle circumference; HGS—handgrip strength; CST—chair stand test; LFI—Liver Frailty Index.

**Table 3:** Comparison of dietary habits between outpatients and inpatients with liver cirrhosis.

Diet Component	Outpatients (*n* = 126)	Inpatients (*n* = 69)	*p* Value
Energy intake (kcal/day)	1604.38 ± 468.75	1547.59 ± 522.35	0.439
Energy intake (kcal/kg/day)	23.15 ± 6.96	21.38 ± 6.84	0.089
Energy deficit	96 (76.2)	63 (91.3)	0.009
Protein intake (g/day)	69.8 ± 24.6	67.72 ± 24.04	0.571
Protein intake (g/kg/day)	1.0 ± 0.36	0.93 ± 0.31	0.183
Protein deficit	77 (61.1)	58 (84.1)	<0.001
Fat intake (g/day)	58.17 ± 21.48	54.01 ± 18.07	0.087
Carbohydrate intake (g/day)	214.59 ± 78.36	201.59 ± 88.93	0.147
Active alcohol intake	11 (8.7)	13 (17.4)	0.073
Alcohol intake (UA/day) *	4.21 ± 1.94	6.24 ± 3.6	0.108

Data presented as mean ± SD or as n of patients and percentages (%). (*) refers only to patients with active alcohol intake.

**Table 4:** Comparison of demographics and clinical characteristics between male and female patients with liver cirrhosis.

Variable	Male (*n* = 136)	Female (*n* = 59)	*p* Value
Age (years)	63.17 ± 10.17	64.36 ± 10.38	0.458
Etiology			0.001
Alcohol	66 (48.5)	19 (32.2)	
Metabolic	28 (20.6)	12 (20.3)	
Viral	38 (28.0)	17 (28.8)	
Others	4 (2.9)	11 (18.7)	
Ascites	51 (37.5)	15 (25.4)	0.102
Varices	54 (39.7)	23 (39)	0.924
HE	6 (4.4)	2 (3.4)	0.741
HCC	16 (11.8)	7 (11.9)	0.998
Presence of co-morbidities	91 (66.9)	36 (61)	0.428
MELD score	12.57 ± 4.29	12.68 ± 5.15	0.882
MELD Na score	14.12 ± 5.07	14.15 ± 6.24	0.967
CTP score	7.01 ± 1.68	7 ± 2.02	0.974
Child–Pugh class	A: 70 (51.5)	A: 27 (45.8)	0.611
	B: 52 (38.2)	B: 27 (45.8)	
	C: 14 (10.3)	C: 5 (8.4)	

Data presented as mean ± SD and median (range) or as n of patients and percentages (%). Abbreviations: HE—hepatic encephalopathy; HCC—hepato-cellular carcinoma; MELD—model for end-stage liver disease; MELD-Na—model for end-stage liver disease-sodium; CTP—Child–Turcotte–Pugh.

**Table 5:** Comparison of anthropometry and liver frailty components between male and female patients with liver cirrhosis.

Variable	Male (*n* = 136)	Female (*n* = 59)	*p* Value
BMI (kg/m^2^)	26.27 ± 5.43	26.91 ± 7.58	0.559
			
<18.9 kg/m^2^	5 (3.7)	7 (11.9)	
18.9–24.9 kg/m^2^	57 (41.9)	20 (33.9)	0.083
>24.9–29.9 kg/m^2^	42 (30.9)	14 (23.7)	
>29.9 kg/m^2^	32 (23.5)	18 (30.5)	
MUAC (cm)	30.79 ± 4.51	32.33 ± 7.29	0.240
TSF (mm)	13.2 (21–46.9)	20.5 (7.8–38.2)	0.002
MAMC (mm)	247.85 (183.24–355.87)	225.5 (175.8–370.68)	0.512
Sarcopenia (SARC-F score ≥ 4)	52 (38.2)	38 (64.4)	<0.001
LFI score	4.45 ± 0.91	4.77 ± 0.88	0.034
HGS (kg)	27.82 ± 11.3	15.4 ± 7.63	<0.001
Low muscle function	59 (43.4)	36 (61)	0.024
CST (s)	12.13 (4.18–40)	12.95 (7–32.8)	0.562

Data presented as mean ± SD and median (range) or as n of patients and percentages (%). Abbreviations: BMI—body mass index; MUAC—mid-upper arm circumference; TSF—triceps skinfold; MAMC—mid-arm muscle circumference; HGS—handgrip strength; CST—chair stand test; LFI—Liver Frailty Index.

**Table 6:** Comparison of dietary intake between male and female patients.

Variable	Male (*n* = 136)	Female (*n* = 59)	*p* Value
Energy intake (kcal/kg/day)	22.6 ± 6.72	22.73 ± 6.96	0.91
Energy deficit	118 (86.8)	41 (69.5)	0.004
Protein intake (g/kg/day)	0.98 ± 0.33	0.99 ± 0.36	0.862
Protein deficit	101 (74.3)	34 (57.6)	0.021

Data presented as mean ± SD or as n of patients and percentages (%).

**Table 7:** Factors associated with hospitalization in patients with liver cirrhosis.

Variables	Univariate Regression Analysis		Multivariate Regression Analysis	
	OR (95% CI)	*p* Value	OR (95% CI)	*p* Value
Age (years)	0.97 (0.94–1.0)	0.08		
Sex (M:F)	1.36 (0.71–2.62)	0.35		
Etiology	0.75 (0.41–1.38)	0.35		
Presence of co-morbidities	1.097 (0.59–2.03)	0.76		
HCC	16.59 (4.72–58.39)	<0.001		
MELD score	1.24 (1.15–1.25)	<0.001		
MELD Na score	1.22 (1.12–1.32)	<0.001		
CTP score	2.14 (1.63–2.79)	<0.001	1.67 (1.12–2.49)	0.010
BMI (kg/m^2^)	0.92 (0.87–0.97)	0.003		
MUAC (cm)	0.90 (0.83–0.98)	0.017		
TSF (mm)	0.93 (0.88–0.99)	0.038		
Sarcopenia (SARC-F score)	1.11 (0.62–1.99)	0.729		
LFI score	2.29 (1.57–3.36)	<0.001	2.73 (0.71–0.91)	0.011
Low muscle function	0.67 (0.37–1.21)	0.19		
Protein intake (g/kg)	0.51 (0.21–1.22)	0.132		
Protein deficit	3.35 (1.6–7.01)	0.001	0.27 (0.08–0.88)	0.031
Energy intake (kcal/kg)	0.96 (0.91–1.0)	0.056		
Energy deficit	0.30 (0.12–0.77)	0.012		

Data presented as odds ratio (95% confidence interval). Abbreviations: HCC—hepato-cellular carcinoma; MELD—model for end-stage liver disease; MELD-Na—model for end-stage liver disease-sodium; CTP—Child–Turcotte–Pugh; BMI—body mass index; MUAC—mid-upper arm circumference; TSF—triceps skinfold; LFI—Liver Frailty Index.

## Data Availability

The original contributions presented in this study are included in the article. Further inquiries can be directed to the corresponding author.
